# Supplementary material for: Degradation in parallel-connected lithium-ion battery packs under thermal gradients
Source: Commun Eng. 2024 Jan 4;3:2. doi: 10.1038/s44172-023-00153-5 (PMC10955900; doi:10.1038/s44172-023-00153-5)
Supplement: Supplementary file 2 — Supplementary information [file 44172_2023_153_MOESM2_ESM.pdf]

Degradation in parallel-connected lithium-ion battery packs under thermal gradients
Supplementary information

Max Naylor Marlow[a], Jingyi Chen[a], Billy Wu[a,*]

[a] *Dyson School of Design Engineering, Imperial College London, London, SW7 2AZ, United Kingdom*

1. Supplementary Methods

1.1. Cell Selection

The cells used in this study were Kokam 5 Ah cells with nominal details shown in Table S1.

Table S1: Properties of Kokam SLPB11543140H5 Cell

Cell Identifier	SPLB11543140H5
Anode Chemistry	Graphite
Cathode Chemistry	NMC/LCO
Nominal Capacity	5 Ah
Nominal Energy	18.5 Wh
Maximum Continuous Discharge Rate	30 C
Maximum Pulse Discharge Rate	50 C
Maximum Continuous Charge Rate	2 C
Upper Voltage Limit	4.2 V
Lower Voltage Limit	2.7 V
Nominal AC Impedance	3 mΩ
Operating Temperature Range	-20 °C - 60 °C
Weight (Nominal)	132 g
Width (Nominal)	43.0 mm
Length (Nominal)	142.5 mm
Thickness (Nominal)	11.7 mm
Volume (Nominal)	0.072 m^3
Gravimetric Energy Density (Nominal)	140 Wh/kg
Volumetric Energy Density (Nominal)	256 Wh/L

In order to group the cells, the capacity and series resistance were considered for grouping. As shown in Figure S1 b, there was a marginally higher resistance group of six cells, and these cells were discarded from selection. The next highest resistance cell was then removed to leave twelve cells to produce six 1S2P packs, and the cells were then sequentially grouped by their corresponding R_0 values to leave six pairs, shown in Figure S1 e-j. As can be seen, the resultant deviations in both capacity and impedance were small, with all resistance differences minimised to below 0.45 % (5.72 $\mu\Omega$) and capacity differences to below 0.85 % (42.3 mAh), excepting pair 3 which showed a larger deviation of 1.314 % (65.6 mAh). Despite this, the spread at SoL

*Corresponding author. Tel.:+44 (0)20 7594 6385
Email address: billy.wu@imperial.ac.uk (Billy Wu)

was deemed acceptable and the cell pairings were subsequently used as shown. To compare to typical values of variation in resistance and capacity found in typical commercial cells, Rumpf et al. [1] assessed the statistical parameter variation in a two batches of cells consisting of a total of 1100 total commercial cells, which is useful to provide typical values. For directly comparable metrics (constant current discharge capacity and R_0) percentage variations of 0.41 - 0.48% and 0.73 - 1.82% respectively between the two batches were found. In this work it was judged that minimisation the resistance variation was essential to reduce any effects on pack degradation induced by more significant differences in cell resistances, and thus pairs were optimised to minimise this variation.

Of the remaining cells from the batch, the group of 4 cells with approximately 5 Ah capacity and 1.31 mΩ R_0 were used for single cell baseline testing, and the remaining lowest R_0 cell was set aside to be used to provide an EIS baseline over a full temperature range, as based on the single spectra EIS results shown in Figure S1 a, the impedance response of all cells from the batch at SoL was highly consistent. To provide a baseline cell for comparison during cell disassembly, a final cell was selected and torn down for imaging.

1.2. Test Bench Development

In order to apply a desired thermal gradient to the cells within the packs, by controlling the thermal boundary conditions, it was required to directly control the surface temperature of the cells. This was accomplished using Peltier elements to either cool or heat an aluminium heat distribution plate which was in direct contact with each broad face (top and bottom) of the cell. The use of heat distribution plates of high thermal conductivity ensures that a homogenous temperature is applied to the cell surface, and similar designs (of which a schematic cross-sectional diagram and CAD image are shown in Figure S2 a and b) were used for both the 1S6P and 1S2P test benches. In both cases, the upper cooling plates were removable to allow insertion of cells as required, using 4 M4 screws. In all cases, the screws were torqued to 0.75 Nm to ensure consistency of the thermal contact between the cell and heating plate. While either thermal paste or thermal interface material would have improved the thermal contact between the plates and cell surfaces, this was not used to simplify the setup procedure of the test benches, an important consideration given the test bench was designed such that cells could be removed regularly for interim characterisation.

Peltier elements have been previously used by researchers in the Imperial College Electrochemical Science and Engineering group to produce a variety of thermally controlled test benches to probe performance of LIBs under a range of thermal conditions [2, 3, 4]. Two versions of the Peltier control system were used in this work. As the Peltier elements operate at a relatively low efficiency, and in the case of cooling, the cell will heat on the reverse side, a method for rejecting this waste heat was required. To accomplish this, a water-cooling circuit was used in both test benches, with a chiller providing cool (15 °C) deionised water to the aluminium water blocks which were attached via a thermal interface compound to the elements.

For the 1S2P test bench, power was supplied via a custom H-bridge motor controller designed and built for control of Peltier elements. In order to control the temperature of the system a Proportional Integral Derivative (PID) control scheme was implemented and programmed in a custom applet in C#. The PID coefficients were tuned using the Zeigler-Nicols method to estimate parameters for a no-overshoot controller. The temperature at the centre of each heat distribution plate was measured using a single K-Type thermocouple, situated at the centre of the cooling plate, using PicoLog TC-08 data logger. The applet received temperature data from the TC-08 and calculated the required heating or cooling power based on a setpoint in

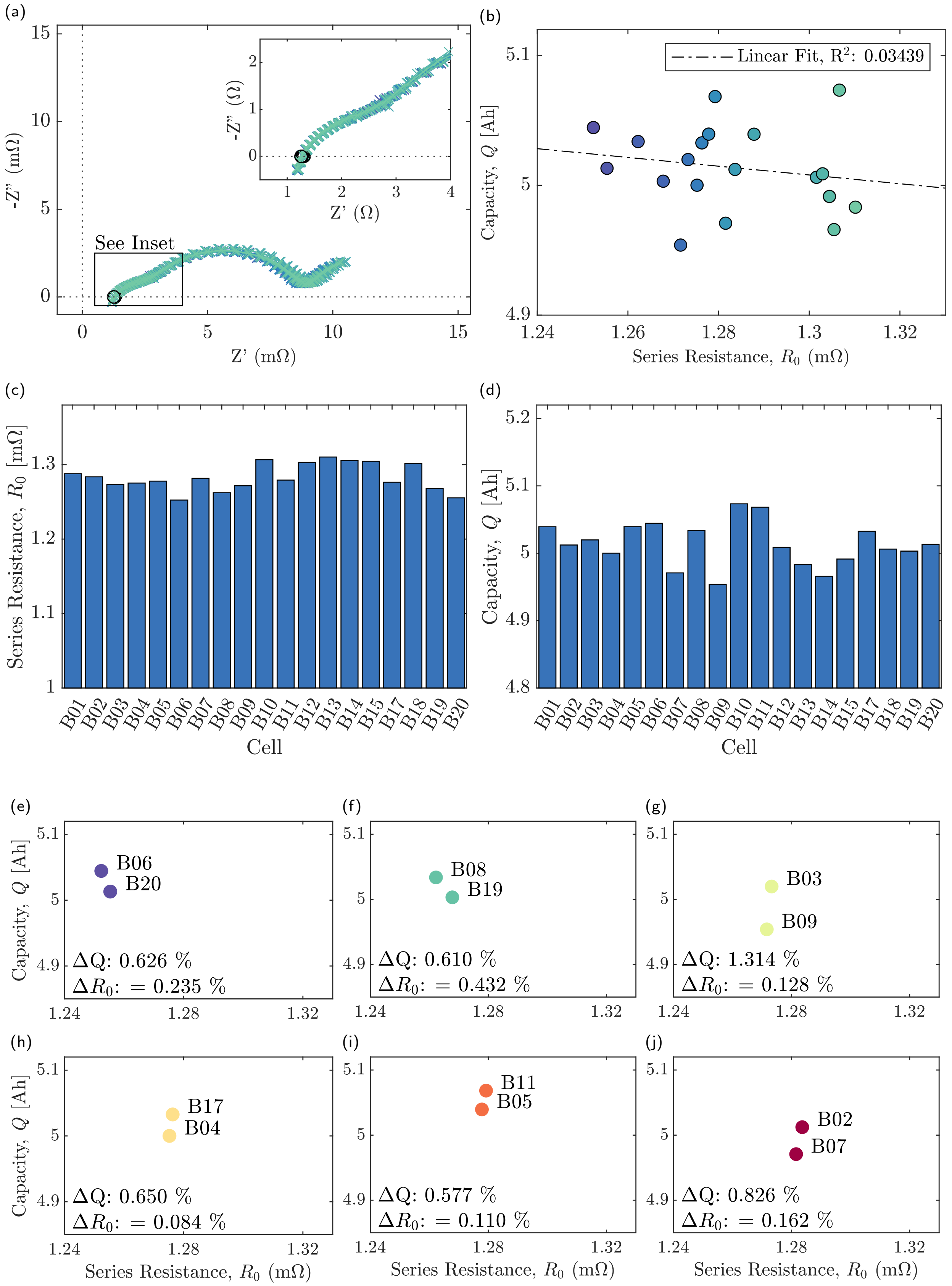

Figure S1: Measured capacity and impedance of cell batch, and allocated cell pairs for 1S2P aging experiments, showing ΔR and ΔQ. a) Cell impedance at 50 % SoC, 20 °C for all 19 cells. b) Resistance and capacity scatter plot for all 19 cells. c) Fitted R_0 values for all cells. d) Measured capacity for all cells at 0.04 C. e) Pair 1. f) Pair 2. g) Pair 3. h) Pair 4 i) Pair 5. j) Pair 6.

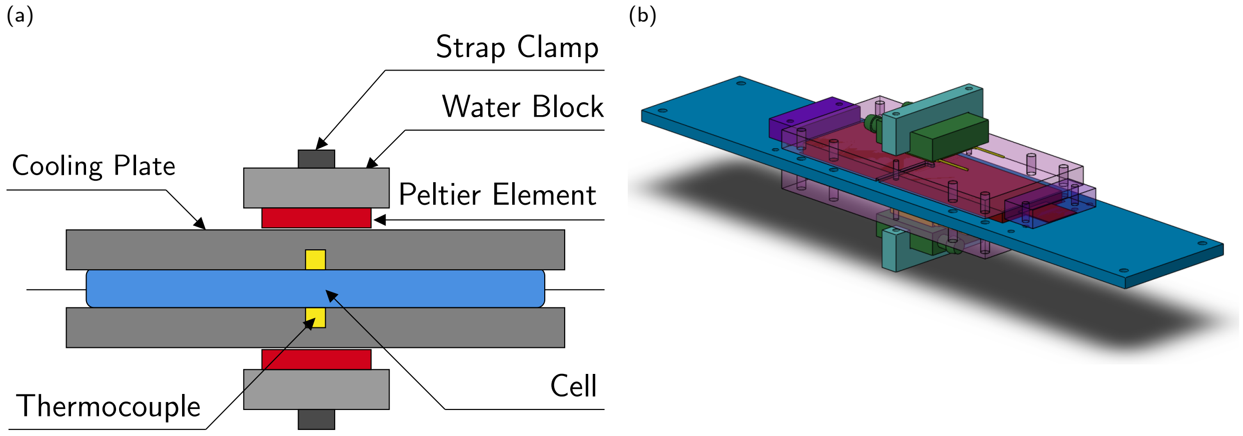

Figure S2: Schematic representation of Peltier surface cooling assembly. a) Cross-sectional schematic of assembly. b) 3-D CAD representation of surface cooling assembly

the software, which was then sent via a serial connection to an Arduino Mega 2560 in the control box. This microcontroller in turn provides an enable signal, direction and pulse width modulated power signal to each slave motor controller board. This system could maintain a controlled setpoint temperature to within $\pm$ 0.10 °C at the temperature measurement point over the required operating range (20.0 – 45.0 °C) with applied C-rates of up to 3 C.

In order to carry out multiple degradation experiments simultaneously, a test bench was developed for testing 6 1S2P parallel cell strings. PCBs were designed and used as busbars with inserted interconnection and current sense resistors. As it was essential to be able to rapidly change the cells, as it was intended to characterise cells from the packs individually during degradation, toggle clamp retained contact blocks were used to make cell connections, and the PCB was soft-gold plated to reduce contact resistances. Typically contact resistances measured 200-250 $\mu\Omega$ and so in all cases contact resistance was measured using a Keithley 2182A Bench Digital Multimeter to ensure consistency of contact resistance between runs and cells within the pack.

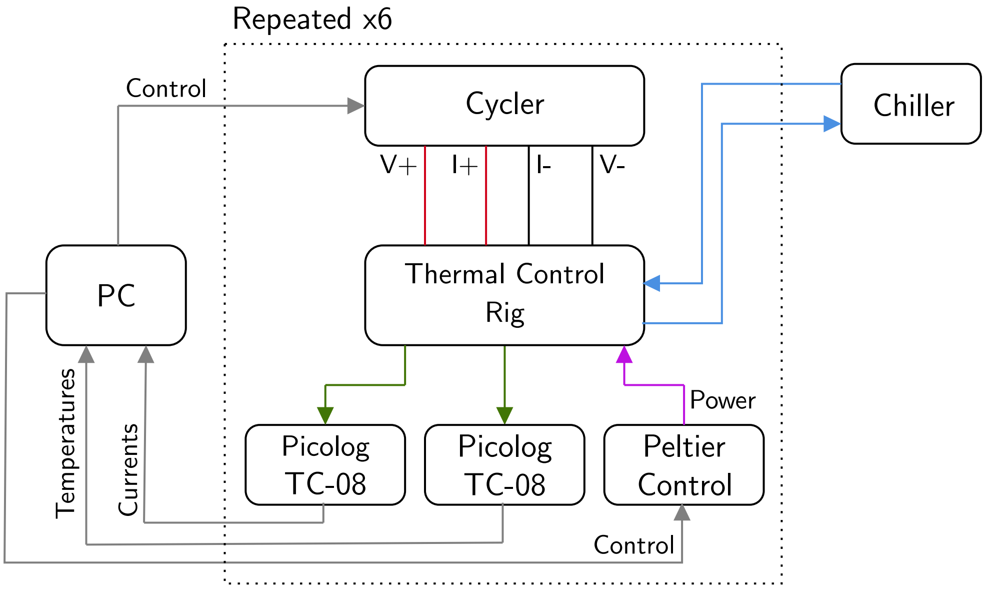

Figure S3: Schematic representation of 1S2P surface cooled test bench

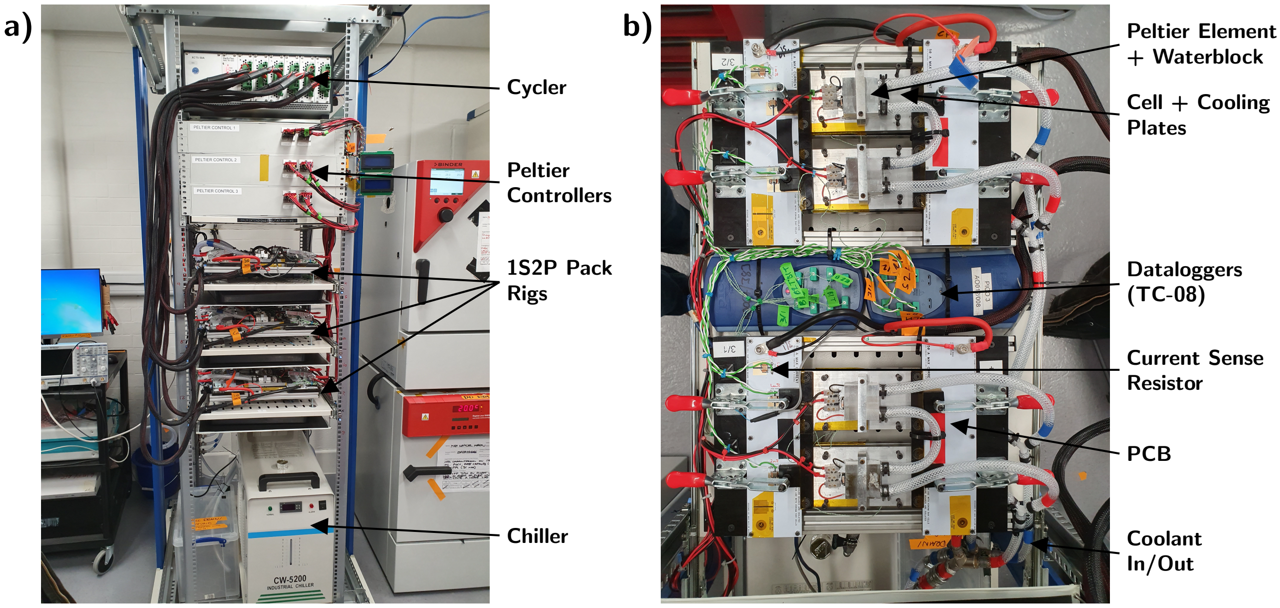

Figure S4: Annotated images of 1S2P pack testing rig. a) Full system. b) Layout of 2 pack fixtures on a rack shelf

A schematic layout of the 1S2P pack test bench is shown in Figure S3. In this configuration, currents and temperatures are logged using TC-08 dataloggers. PID control is implemented in an applet running on the PC, which reads the temperatures and then serially communicates the desired control point to the Peltier Controller, with an Arduino Mega 2600 handling onboard control. Figure S4 a shows an image of the overall configuration of the pack test bench, configured to test 6 1S2P packs. As can be seen, the test bench was built in a rack cage, with the battery cycler (BaSyTec 50 A XCTS G1), PC and custom Peltier controllers at the top, and the water chiller at the bottom of the rack. The individual pack test fixtures were distributed between on three full depth pull out shelves, with two fixtures per level, as shown in Figure S4 b. Each shelf contained both the test fixtures and PicoLog TC-08 dataloggers used for current and temperature sensing and was designed to be self-contained. The levels were separated by 1 mm Mild Steel sheeting to provide isolation in the event of cell failure and fire.

In the 1S2P test bench, cooling water was provided by a CW-5200 Chiller with an internal pump. The coolant, deionised water, was distributed to each level of the rack via quick disconnect fittings to allow rapid maintenance and switchover of cells. The pipework was designed to ensure a similar length flow path through each individual pack test fixture, to ensure sufficiently low temperature coolant was available for heat rejection from each Peltier element. A spill tray was attached below each level to ensure any coolant leaks would be collected and returned to the sump at the bottom of the test bench.

Both test benches required calibration of resistances and sensing in order to ensure accurate measurement of currents, and to ensure consistency between modelling and experimental results. Current sensing within the parallel cell strings was accomplished using current shunt resistors to measure the individual string currents, as well as the total applied current to the pack. Current sense resistors with a nominal value of 1 mΩ and 2 mΩ were used (Bourns CSS4J-4026R-1L00F and CSS4J-4026R-2L00F respectively) with a nominal 1 % accuracy. These current sense resistors were in part selected due to their 4-wire configuration allowing for increased measurement accuracy alongside their low temperature coefficient of 75 ppm/°C, which ensures that resistive

heating of the current sense resistors does not lead to drift in current measurements.

In both cases, once assembled, the resistances of the busbar boards and current sense (or shunt) resistors were measured using a potentiostat (Biologic VMP-3) to apply a current over the current sense resistors, and the voltage drop over the current sense resistors was measured using the sense cables attached. A linear voltage sweep between the working and counter electrodes, slewing at a rate of 2500 mV/s between 0 and 10 mV (selected to give a peak current of approximately 10 A, the maximum allowable current that could be applied with the VMP-3). The true resistance of the current sense resistors was calculated by fitting a straight line to the current response, meaning that the gradient of the line gave the resistance of the cell, by Ohms law. For calibration of various resistances throughout the busbar the same approach was used, measuring the response between marked points on the busbars.

Once calibrated, the voltage drop across each current sense resistor was recorded in the 1S2P test bench using a PicoLog TC-08 data logger, which has a 20-bit analogue-to-digital converter, therefore giving a voltage resolution of 67 nV in the $\pm$ 70 mV range. In the 1S6P test bench, a PicoLog ADC-24 datalogger was used, with a 24-bit analogue-to-digital converter, giving a voltage resolution of 2.3 nV in the $\pm$ 39 mV range. Given the current sense resistor values and expected operating currents this is an adequate resolution to ensure accurate recording of cell currents.

In order to ensure that the cell surface temperature was controlled to the desired setpoint, a calibration cooling plate was manufactured with 4 additional thermocouple slots milled into the surface. K-type thermocouples were embedded into this plate using thermal epoxy, at locations shown in Figure S5 a, and the plate was setup in one of the 1S2P pack test fixtures. A cell was introduced and cycled at 2 C to test the uniformity of cell surface temperature with a heat-generating cell introduced. After the setpoint was set to 20 °C and allowed to settle, a 600 s discharge followed by a 600 s charge was applied. The setpoint was then moved to 45 °C and the process repeated.

Figure S5 b shows the temperature at each measured location and the applied current. It is expected that the outer measurement points will be at a slightly lower temperature, as the Peltier element is both situated centrally, leading to some convective temperature loss towards the edges of the plate. Additionally and the control sensor used is $\mathrm{T_M}$, which will also lead to somewhat lower temperatures further from the plate midpoint. As can be seen in Figure S5 c, the heating plate performed well, with a maximum difference between measurements (typically between $\mathrm{T_{R,O}}/\mathrm{T_{L,O}}$ and $\mathrm{T_M}$) of 0.82 °C at a setpoint of 20 °C, and a maximum difference of 1.26 °C at a setpoint of 45 °C. The plate was also moved over the entire temperature range used in the study (20-45 °C) and rested at each setpoint for 600 s to evaluate the system settling time (Figure S5 d). The maximum difference was again found to occur at a setpoint of 45 °C, due to the increased temperature difference between the plate and ambient air leading to increased convective losses. The maximum temperature difference in this case (Figure S5 e) was found to be 1.27 °C. As the magnitude of thermal gradients under investigation was an order of magnitude greater than the maximum temperature differences measured, including under typical cycling loads, the surface cooling test fixtures were deemed sufficiently accurate for use. While some overshoot in the PID control was also seen, this was not deemed an issue as all planned measurements were at steady-state temperatures.

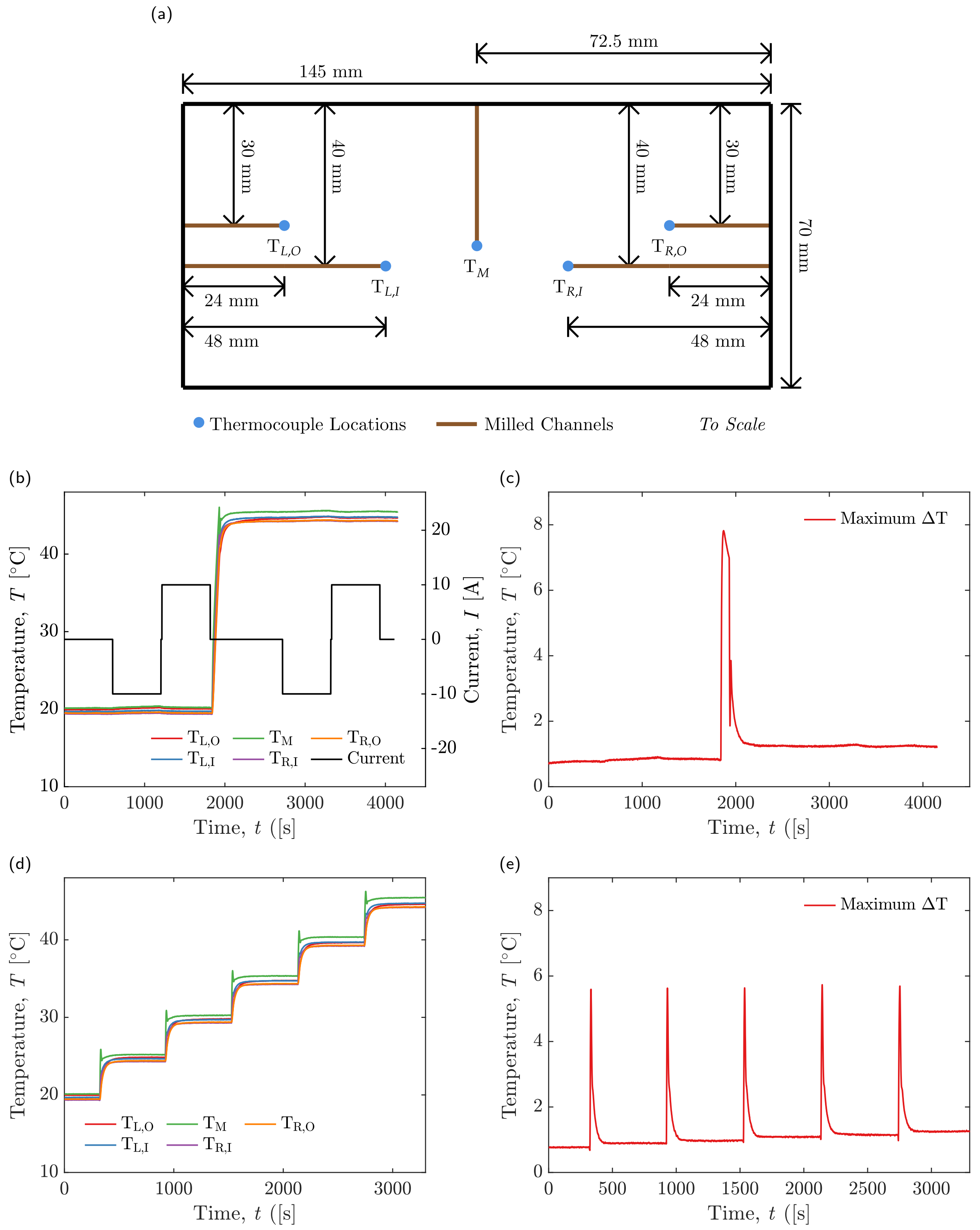

Figure S5: Calibration temperature results for surface cooling fixture. a) Schematic showning the location of the additional thermocouples used during calibration. b) Calibration temperature under 2 C load, charge and discharge. Initial setpoint = 20 °C. setpoint moved to 45 °C at t = 1800 s. c) Calibration temperature residual under 2 C load. e) Calibration temperatures under no load, with temperature setpoint steps of 5 °C. e) Calibration temperature residual under no load.

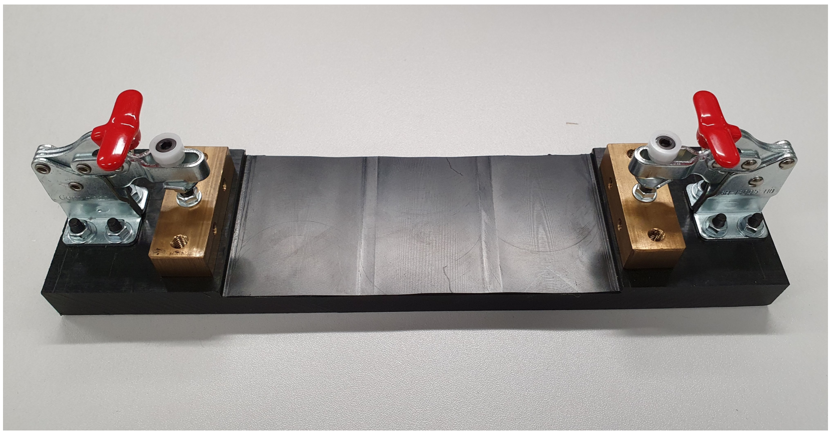

Figure S6: Images of single cell test fixtures used.

2. Supplementary discussion

2.1. *Cell Capacity Loss*

The absolute measured static capacity values corresponding to those presented normalised in Figure 2 (c) and (d) can be found in Figure S7.

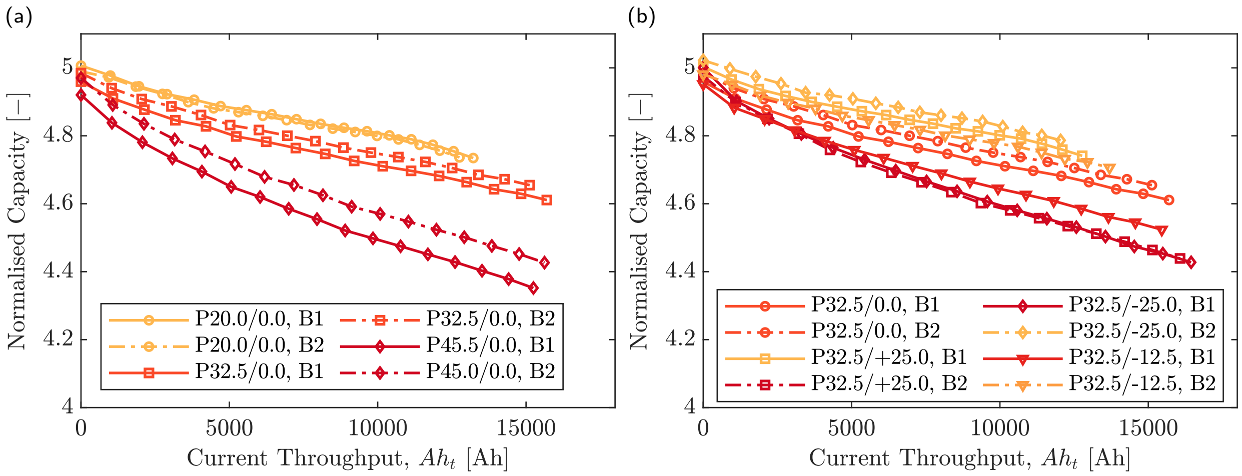

Figure S7: Static measured cell capacity (at 0.1 C, 20 °C) versus current throughput for all homogeneous temperature (a) packs and heterogeneous temperature (b) packs. Note that in c and d, trace colour is proportional to cell cycling temperature

2.2. *Pack State of Charge Deficit Estimation*

Within parallel strings, accessible capacity is reduced by the EoD SoC deficit, i.e. remaining unused cell capacity at EoD. Figure 8 shows the SoC deficit at EoD for all packs tested in this work. In order to calculate the SoC deficit at the end of each discharge half-cycle, the underlying cell static capacity is required at each cycle. In this work, the static capacity for each individual cell is measured at 0.1 C at 20 °C every 125 cycles. This allows the SOC deficit is calculated for each pack cycle, by subtracting the measured charge passed from the static capacity of the cells on each cycle. The static capacity of the cells is estimated by using a spline interpolation of the individual cell capacity loss data. aAs shown in Figures 2 and S7 the degradation observed is at no point significantly non-linear over the cycle number range studied, and thus this interpolation

gives a good estimate of the static capacity of each cell during cycle aging and thus is therefore used to estimate the SoC deficit.

2.3. Diagnostic Model

In order to use the cell OCV response as a quantitative as well as qualitative diagnostic tool, it was necessary to fit a model for the initial behaviour of the cell, which then allows predictions to be made about OCV behaviour over the lifetime of cells, and for quantitative measurements of cell degradation modes to be obtained. In order to achieve this goal, it was necessary to fabricate half cells to obtain reference measurements of the half-cell voltage response, estimate the initial loading ratio and anode/cathode offsets and fit the model. This allowed for both the prediction of the qualitative changes in cell OCV response over the life of the cell alongside quantitative fitting of the model to identify the proportion of combined degradation modes occurring in an aged cell. Dubarry et al. [5] highlighted the complementary nature of ICA and DVA analysis, as whilst capacity quantification is clearer using DVA, using ICA it is simpler to decipher degradation modes. However in this work an OCV model was selected for use, derived from work by Birkl et al. [6, 7].

2.3.1. Cell Disassembly, Coin Cell Fabrication and p-OCV Measurement

To obtain an accurate fit to the OCV curves from full cell measurements, reference half cell curves were required to identify the individual voltage response of each electrode. Therefore, a fresh 5 Ah Kokam cell was discharged to 2.7 V and disassembled in an Argon-filled glovebox to provide sample electrodes for coin cell fabrication. The cell was opened using a ceramic scalpel to cut the pouch, and after opening, the cathode and anode were gently separated and the cell tabs cut. The cell stack was unwound as required to provide electrodes to be harvested into coin cells. In the Kokam cell, all but the outer layers were coated on both sides, and therefore it was necessary to remove the electrode material coating one side of both anode and cathode sheets. This was achieved by wetting with 1-Methyl-2-Pyrrolidinone (NMP, Sigma-Aldrich), and gently wiping the electrode to dislodge the active material. Once single sided electrodes were obtained, circular discs of 16 mm diameter were punched out and rinsed using 1 M dimethyl carbonate (DMC, Sigma-Aldrich), before vacuum drying at 45 °C for a minimum of 1 hr to remove any remaining DMC. The as-prepared electrodes were assembled into CR2032 form-factor coin cells with lithium anodes (0.45mm thickness, 15.6 mm diameter), a polypropylene separator (Celgard 2400, 25 μm) and filled with 100 μL of 1.0 M $LiPF_6$ in a 1:1 mix of ethylene carbonate/DMC (Sigma-Aldrich). The cells were then crimped and allowed to rest overnight before testing, in order to ensure that electrodes were fully wetted. 8 sample coin cells from both the anode and cathode were prepared to ensure consistency in 2 separate batches.

Once half-cells were been fabricated, the p-OCV response of each half cell was measured using a 10 mA LAND Battery Cycler. In each case, the cells were cycled under a constant current load at C/40, with the current magnitude selected based on the estimated capacity of the anode and cathode, calculated based on the area of the harvested electrodes divided by the approximate total area of anode and cathode in the cell (in this case 0.05 mA). The anode was cycled between 0.01 and 1.5 V, while the cathode was cycled between 4.3 and 3.0 V. Cycling was carried out at room temperature, which was 23 °C $\pm$ 2 °C. Reference measurements from a separate fresh Kokam 5 Ah cell, from the same batch, were carried out by discharging the cell at C/20 (0.25 A) using a BaSyTec XCTS G1 Battery Cycler. The cell was initially charged at 1 C to 4.2 V using a CC-CV protocol with a cutoff current of C/100 (0.05 A), which was followed by a 1 hr rest period and subsequent C/20 discharge. All measurements on the full cell were carried out in

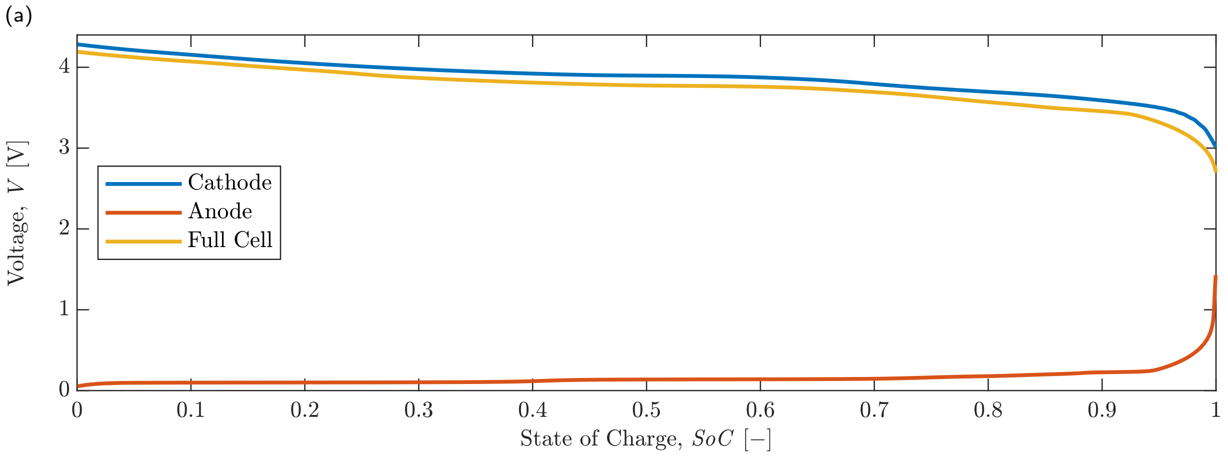

Figure S8: Anode, cathode and reference full cell OCV curves, measured at C/40 (half cell) and C/20 (full cell).

an incubator (Binder KB53) set to 20 °C. The reference voltage measurements against SoC are shown in Figure S8.

2.3.2. Parameterising the OCV Model

As can be seen in Figure S8 a, voltage plateaus exist in the OCV response of each electrode, which occur during phase transitions in the cathode [8] or staging in the graphite anode [9] during lithiation or delithiation. Typically, in commercial LIBs, the anode capacity is higher than the cathode capacity, due to the differential loading ratio of the electrodes, while the overall capacity of both electrodes is typically higher than can be accessed safely in a full cell due to both the electrolyte stability window and the need to suppress lithium plating in the anode. Additionally, there is typically an offset found between the SoC of each electrode. In order to identify the capacity and offset of each electrode, an OCV model based approach, derived from work by Birkl et al. was selected [6, 7] due to its simplicity and ease of parameterisation.

Broadly the OCV model functions by adjusting the half cell measured p-OCV responses to minimise the error between the measured cell OCV response and the simulated OCV response based on a given alignment of half cell OCV curves. This allows identification of electrode capacities and offsets for a given cell SoH, and therefore by comparing the identified cell parameters at SoL and EoL it is possible to identify the degradation modes occuring within the cell. This approach differs from that taken by Birkl et al. [7] in that the measured half cell curve is used directly, rather than via fitting a parametric function to the half cell curves as described in [6]. This approach is not appropriate for on-board applications, due to the increased computational effort and memory required, however for offline diagnostics this is adequate. A downside of this approach is it does not allow adjustment of the half cell OCV curves based on degradation specific to certain electrode phases, although in Birkl et al.'s work it is assumed that the relative shape of each half cell OCV curve also does not change.

For a LIB, each cell SoC value corresponds to a given lithiation fraction (denoted z) for both the anode and cathode. As the degree of lithiation in a given electrode increases, the voltage of the electrode decreases. However, the cell voltage range is fixed in order to ensure stable and safe operation, and therefore each electrode may not be fully lithiated or delithiated when the cell is either fully charged or discharged. By adjusting the lithiation limits the shape of the simulated full cell OCV curve may be adjusted while the capacity of the simulated full cell remains constant. The relationship between z, the anode and cathode OCV and full cell OCV are shown in Figure

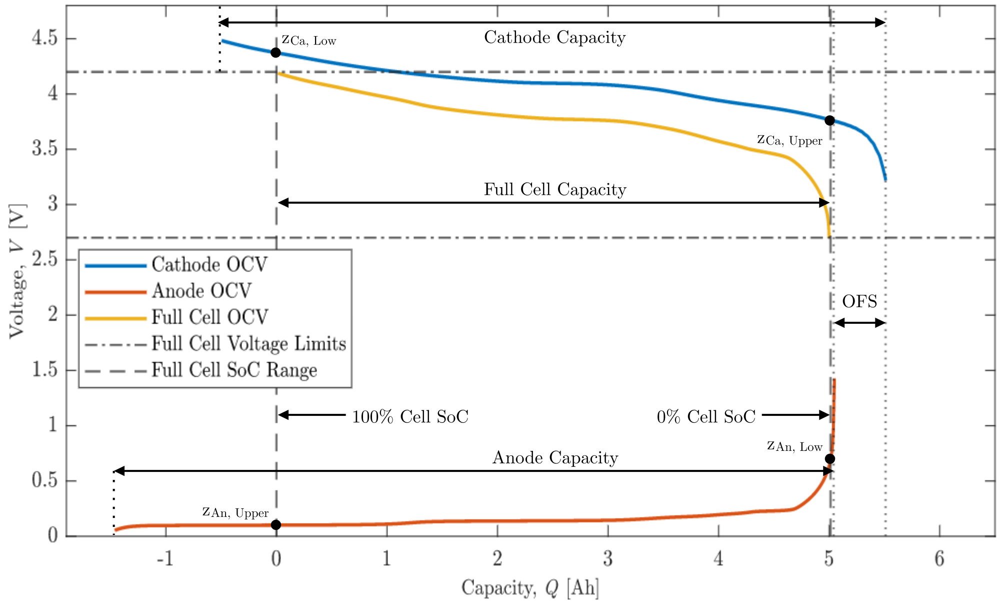

Figure S9: Explanatory schematic showing half cell alignment and full cell OCV. The maximum and minimum lithiation state of each half cell at 0 and 100 % full cell SoC, z is marked. Note that half cell voltage curves are adjusted for clarity and are not presented to scale in this figure.

S9.

As shown in Figure S9, over the full cell SoC range when the cell is fully charged (100 % SoC) the cathode is delithiated to the maximum extent possible within the defined cell operating voltage range. Meanwhile the anode is again lithiated to the maximum extent possible within the given voltage range. It is not desirable to either fully delithiate the cathode, in order to maintain stability of the cathode, or to fully lithiate the anode, to avoid the anode potential becoming negative and inducing lithium plating. Therefore, typically excess capacity is available at SoL in both the anode and cathode. At SoL for a given anode and cathode half cell OCV response, the lithiation fraction for the cathode over the full cell voltage window ranges from $z_{Ca,Low}$ at 100 % full cell SoC to $z_{Ca,Upper}$ at 0 % full cell SoC. The corresponding values for the anode are $z_{An,Upper}$ at 100 % full cell SoC to $z_{An,Low}$ at 0 % full cell SoC.

Formally the model can be divided into two sections. Firstly and critically, the OCV fitting process is described as follows. Based on two half cell voltages, the full cell predicted voltage is given by Equation 1. The quantitative degradation mode fitting is described in 2.3.4.

$$V_{Full}\,(SoC) = V_{Cathode}\,(z|_{SoC}) - \; V_{Anode}\,(z|_{SoC}) \tag{1}$$

Where $V_{Full}(SoC)$ is the full cell voltage as a function of full cell SoC, $V_{Cathode}\,(z|_{SoC})$ is the cathode voltage as a function of the cathode lithiation fraction at a given full cell SoC and $V_{Anode}\,(z|_{SoC})$ is the anode voltage as a function of the anode lithiation fraction at a given full cell SoC. The vector of half cell lithiation fractions is defined by Equation 2.

$$z = [z_{Ca,\;Low},\; z_{An,\;Upper},\; z_{Ca,\;Upper},\; z_{An,\;Low}] \tag{2}$$

Subsequently the model parameters are estimated by minimising the objective function defined in Equation 3.

$$\arg_z \min \text{RSME} = \sqrt{\frac{\sum_i^n (\hat{V}_{Full,\ i}(z) - V_{Full,\ i})^2}{n}} \tag{3}$$

Where $\hat{V}_{Full,\ i}(z)$ is the simulated cell OCV at measurement point i for a given value of z, $V_{Full,\ i}$ is the measured cell OCV at measurement point i, and n is the number of measurements. The error between the simulated and measured OCV is minimised in MATLAB 2021a, using the '*lsqcurvefit*' function and trust-region-reflective minimisation algorithm. After minimisation, the vector z is evaluated to extract the individual electrode capacities and offset, using Equations 4 - 6.

$$C_{An} = \frac{C_{Cell}}{z_{An,Upper} - z_{An,Low}} \tag{4}$$

$$C_{Ca} = \frac{C_{Cell}}{z_{Ca,Upper} - z_{Ca,Low}} \tag{5}$$

$$OFS = (1 - z_{Ca,Upper}) \cdot C_{Ca} - z_{An,Low} \cdot C_{An} \tag{6}$$

Where C_{An}, C_{Ca} and OFS define the anode capacity, cathode capacity and offset respectively. These parameters define the cell capacity and stoichiometry at a given SoH value. C_{Cell} is defined as the cell capacity during p-OCV measurements obtained.

Based on this approach for the reference cell at SoL, the initial loading ratio was estimated based on the ratio of adjusted capacity between the anode and cathode half cells and was found to be approximately 1.146 (the cathode capacity ratio was 1.1057 while the anode capacity ratio was 1.267), which matches values of 1.1-1.3 found in the literature [10, 11]. It is expected that the anode capacity will be in excess when the cell is fully charged, in order to suppress lithium plating.

After error minimisation for the reference cell was completed the predicted OCV response of the cell could be calculated and compared to the measured cell OCV response. Predicted and measured cell OCV responses can be seen in Figure S10 a, whilst the voltage residual error is shown in Figure S10 b. While the overall RSME was 21.69 mV, for the bulk of the SoC range predicted the error was found to be below 5 mV, with a large contribution to the error at low SoCs, i.e. below 10 % SoC. This was not viewed as a significant issue as the bulk of phase transitions and staging occur at higher SoCs, and so this error does not affect overall fitting. To avoid biasing the fit towards low SoC values, the final 10 % SoC of the discharge is discarded from the calculation of the RSME, an approach derived from Birkl et al. [7]. This fit ultimately resulted in an RSME of 7.63 mV in the OCV fit at SOL.

2.3.3. Simulating Cell Degradation Modes and Effects

Using the half-cell OCVs obtained from the harvested coin cells, the fitted OCV model may be operated in 'forwards' mode i.e. predicting the changes to the full cell OCV response for given changes to the anode and cathode lithiation fractions at EoC and EoD, and the offset. Active material may be lost either lithiated or delithiated state, so 5 possible degradation modes are available: $\text{LAM}_{\text{Ca, Delithiated}}$, $\text{LAM}_{\text{Ca, Lithiated}}$, $\text{LAM}_{\text{An, Delithiated}}$, $\text{LAM}_{\text{An, Lithiated}}$ and LLI. It is critical to note that whilst in 'forwards' mode, it is possible to simulate loss of lithiated or delithiated active material, it is **not** possible when diagnosing degradation to make this distinction. This is because when fitting to experimental data there is no way to distinguish between loss of lithiated anode/cathode or loss of delithiated anode/cathode combined with LLI, which would

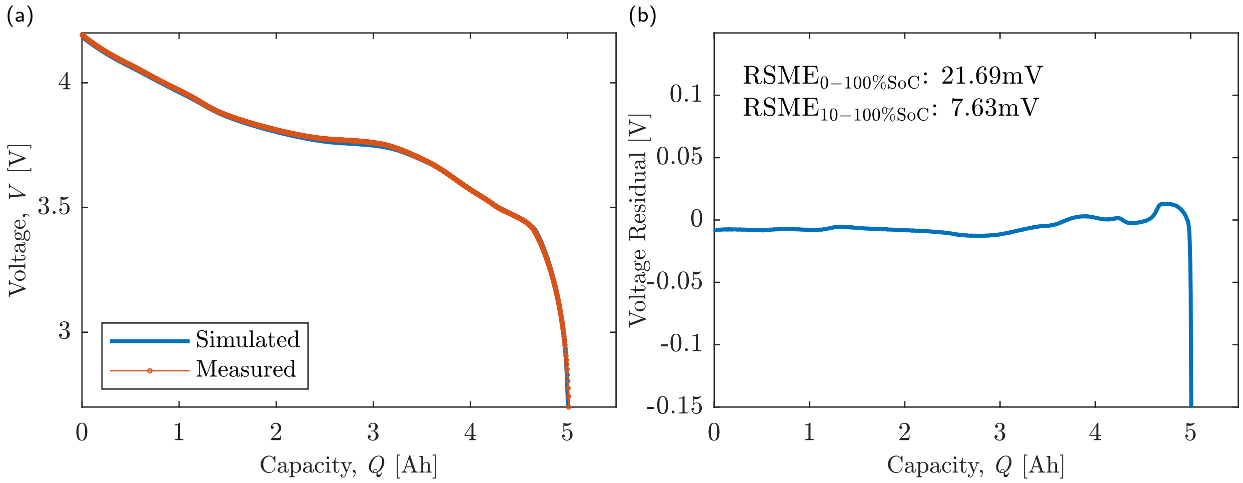

Figure S10: a) Simulated OCV curve and measured p-OCV at C/20. b) Simulated OCV voltage residual and RSME. RSME calculated both over full SoC range and from 10-100 % SoC to avoid bias.

cause the same changes to the full-cell voltage response. Using these simulated translations of the full cell OCV response, it is possible to comment qualitatively on the implications of changes in experimentally derived OCV results. As an example, the changes to the simulated full cell OCV curve are shown in Figure S11, with simulation results for each mode up to 20 % degradation are shown:

- **Loss of Delithiated Anode** – Loss of Delithiated anode material leads to a scaling of the anode half-cell OCV curve, with the EoD position ($z_{An,\ Low}$) fixed (Figure S11 a). Due to the excess anode capacity in the cell, this leads to negligible changes in the simulated OCV response until there is no longer any excess anode, and minimal changes to the full cell capacity. This is due to the fact that the initial cell stoichiometry identifies an approximately 26 % excess capacity in the anode at EoC. Some small changes to the full cell OCV response are identified, with an increased voltage at the anode voltage plateaus at 1.75 and 4.75 Ah, sharpening these plateaus.

- **Loss of Lithiated Anode** – Loss of lithiated anode material leads to a scaling of the anode half-cell curve with the EoC ($z_{An,\ Upper}$) position fixed (Figure S11 b). Qualitatively this leads to a shift to lower capacity values of the anode voltage plateau initially occurring at 4.75 Ah and a significant reduction in overall cell capacity. The voltage at the anode voltage plateaus at 1.75 and 4.75 Ah decreases, reducing the prominence of these plateaus,

- **Loss of Delithiated Cathode** - Loss of delithiated cathode material leads to a scaling of the cathode half-cell curve with the EoD ($z_{Ca,\ Low}$) position fixed (Figure S11 c). Loss of delithiated cathode leads to an overall reduction in cell voltage and a leftwards shift of all cathode voltage plateaus. It is noted that at 10 % simulated degradation, the changes to the cell OCV response is minimal, although in simulations of higher levels of degradation the movement was more significant. This is due to the 10 % excess cathode capacity at EoD leading to minimal changes of up to 10 % loss of delithiated cathode, and implies that limited loss of delithiated cathode will lead to minimal overall capacity loss until this excess capacity is lost.

- **Loss of Lithiated Cathode** - Loss of lithiated cathode material leads to a scaling of the cathode half-cell curve with the EoC ($z_{Ca,\ Upper}$) position fixed (Figure S11 d). This leads

to a movement to lower capacities of the all cathode voltage plateaus and a reduction in overall cell voltage. Unlike in the case of loss of delithiated cathode, a reduction in overall cell capacity is immediately seen, due to the additional LLI occuring

- **Loss of Lithium Inventory** – Loss of lithium inventory is caused by the loss of cyclable lithium due to consumption in irreversible side reactions during SEI formation or being trapped in active material which is lost. LLI leads to a leftwards translation with respect to full cell capacity of the anode half-cell curve (Figure S11 e). This leads to a reduction in the capacity at which anode plateaus occur and an increase in the capacity at which cathode plateaus occur, due to the shifting cell stoichiometry.

In conclusion, using the initial fitting of the model, it is possible to build a set of qualitative distinctions between different degradation modes and their effects on the full cell OCV response, allowing qualitative descriptions of internal cell degradation modes to be made.

2.3.4. Quantitative Degradation Mode Fitting

While understanding the qualitative effects of different degradation modes is helpful, it is of overall limited use, especially when attempting to identify degradation modes when multiple modes are occurring. For example, a cell which is cycled at high temperature and high rate may see loss of active material due to particle fracture in the electrodes, while also undergoing loss of lithium inventory due to high temperatures promoting irreversible side reactions. As such, a quantitative model is needed to assess such contributions to overall cell degradation. Birkl et al. [7] previously extended the parametric OCV model derived in their previous work [6] to identify degradation modes occurring within cells, by fitting to the cell OCV response. The quantitative diagnostic model described here is a derivation of this work.

The approach taken in this work is outlined as follows. By using the parametric OCV model introduced in Section 2.3.2, it is possible to identify the half cell capacities and cell stoichiometry at any given SoH value for a cell. This assumes that both the cell OCV curve shape does not change beyond translation and scaling, and that p-OCV measurements remain an accurate approximation of the true cell OCV at all measured points. The OCV model is used to identify cell parameters at both SoL and EoL, and subsequently the degree of degradation in each identifiable degradation mode ($\mathrm{LAM_{Ca}}$, $\mathrm{LAM_{An}}$ and LLI) may be estimated using Equations 7 - 9 respectively.

$$LAM_{Ca} = 1 - \frac{C_{Ca,\ EoL}}{C_{Ca,\ SoL}} \tag{7}$$

$$LAM_{An} = 1 - \frac{C_{An,\ EoL}}{C_{An,\ SoL}} \tag{8}$$

$$LLI = C_{Ca,\ SoL} \cdot \mathrm{LAM_{Ca}} + OFS_{EoL} - OFS_{SoL} \tag{9}$$

Where $C_{Ca,\ BoL}$ and $C_{Ca,\ EoL}$ are the cathode capacity at SoL and EoL respectively, $C_{An,BoL}$ and $C_{An,EoL}$ are the anode capacity at SoL and EoL respectively and OFS_{BoL} and OFS_{EoL} are the stoichiometric offset at SoL and EoL respectively. It is important to note that whilst there are 5 possible degradation modes within the cell i.e. $\mathrm{LAM_{Ca,\ Delithiated}}$, $\mathrm{LAM_{Ca,\ Lithiated}}$, $\mathrm{LAM_{An,\ Delithiated}}$, $\mathrm{LAM_{An,\ Lithiated}}$ and LLI, the model is unable to distiguish between loss of lithiated and delithiated active material, as the change to the cell OCV curve from an equal contribution of LLI and $\mathrm{LAM_{An/Ca,\ Delithiated}}$ will lead to an identical change to the cell OCV

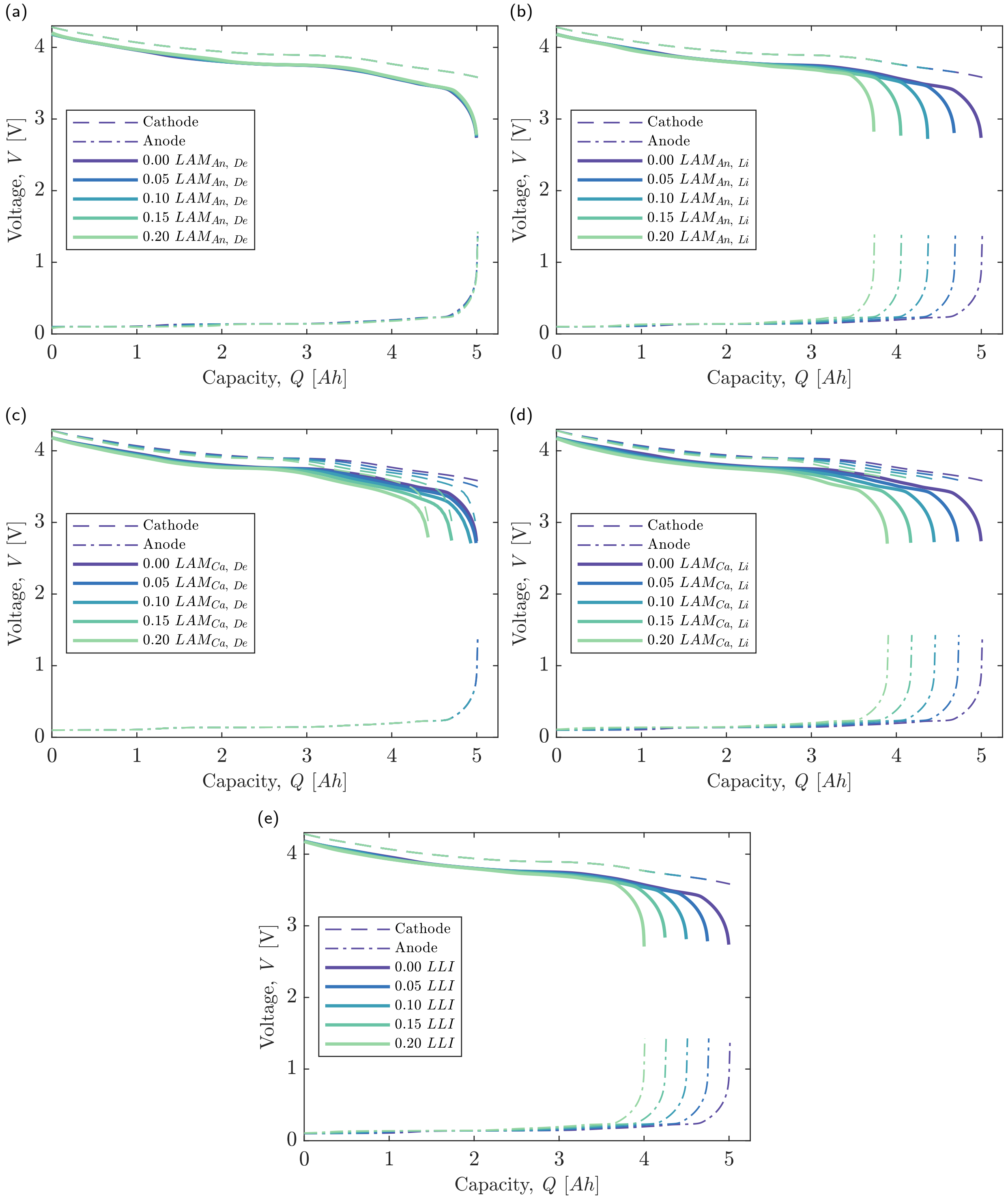

Figure S11: Simulated OCV responses for the the following cases; a) OCV response for 0 - 20 % $\mathrm{LAM}_{\mathrm{An,\ Delithiated}}$. b) OCV response for 0 - 20 % $\mathrm{LAM}_{\mathrm{An,\ Lithiated}}$. c) OCV response for 0 - 20 % $\mathrm{LAM}_{\mathrm{Ca,\ Delithiated}}$. d) OCV response for 0 - 20 % $\mathrm{LAM}_{\mathrm{Ca,\ Lithiated}}$. e) OCV response for 0 - 20 % LLI

response as an equal amount of $\mathrm{LAM}_{\mathrm{An/Ca,\ Lithiated}}$. As such whilst it is possible to simulate (as presented in Section 2.3.3) the differing changes to the cell OCV curve caused by loss of lithiated or delithiated electrode material using the parametric model operating in forward mode, it is not possible to identify if lithiated or delithiated active material is lost from measured cell OCV data. As such only total LAM for each electrode is considered in this analysis.

2.3.5. Validation of Quantitative Diagnostic Model

In order to validate the quantitative diagnostic model, synthetic voltage curves were generated for the Kokam 5 Ah cell under 5 and 10 % loss in each of the 5 degradation modes singly, and for 1, 4 and 10 % of all modes (referred to as 'mixed-mode'). Again it is critical to note that whilst this model may be used in this way to test fitting, distinction cannot be made in practical use between loss of lithiated or delithiated active material. Using identical processes to those used for fitting experimental data the diagnostic fitting model was used to estimate the degree of each degradation mode occurring. Figure S12 a-e summarises the predicted loss in each mode for the 5 and 10 % synthetic single mode losses. For each single degradation mode, the fitting tool is able to accurately identify the dominant degradation mode occurring. In all cases the model is able to diagnose the amount of degradation occuring with <2.5 % absolute error, whilst the diagnosed $\mathrm{LAM}_{\mathrm{Ca}}$ is accurate to <0.25 % in all cases. Performance in identification of $\mathrm{LAM}_{\mathrm{An}}$ and LLI is worse, as generally the anode voltage curve is flat. This means the fitting algorithm is biased towards identification of changes to the cathode voltage curve rather than the anode, as a change in lithiation state of the cathode will generally lead to a greater change in full cell voltage. However, in all cases the model is able to correctly identify the dominant degradation mode, and performs sufficiently accurately in identification of the amount of each mode.

Figure S13 a-c summarises the predicted loss in each mode for the 1, 4 and 10 % synthetic mixed mode scenarios. In this case again the model is able to correctly identify that both all degradation modes are occurring, and reasonably accurately predict the amount of each mode occurring. The absolute error is <3.5 % in all cases, with typical relative errors in prediction of <10 %. As in practice it is likely that a LIB will undergo multiple simultaneous degradation mechanisms, leading to occurrence of multiple degradation modes, validation in a mixed mode scenario is critical, and indicates that the model performs adequately in quantification of degradation.

2.3.6. Diagnostic Model Overview

To summarise, the models and tools introduced in this section, firstly a model for simulating the effects of 5 identified degradation modes on the OCV curve for the Kokam 5 Ah cell is presented and validated, using half-cell OCV curves to construct the full cell response. The model parameterisation was possible using the OCV model fitting approach pioneered by Birkl et al. [6]. Using this model to fit to experimental data for a full cell, the excess anode and cathode capacity alongside the loading ratio and offsets may be estimated. Secondly, a quantitative diagnostic fitting methodology is presented and validated. The diagnostic tool presented is able to both correctly and accurately identify the dominant degradation mode from single-mode synthetic OCV curves. The diagnostic tool has also been shown to be able to accurately identify mixed-mode degradation, excepting some inaccuracy in identification of $\mathrm{LAM}_{\mathrm{An,\ Delithiated}}$. Using these tools it is possible to carry out a detailed analysis of the degradation modes occurring within parallel cell strings.

2.4. Cell Impedance Measurements and Fitting

In order to investigate changes to, EIS measurements were taken at the SoL and EoL for each cell at 50 % SoC with respect to measured cell capacity at SoL and EoL. In all cases, the

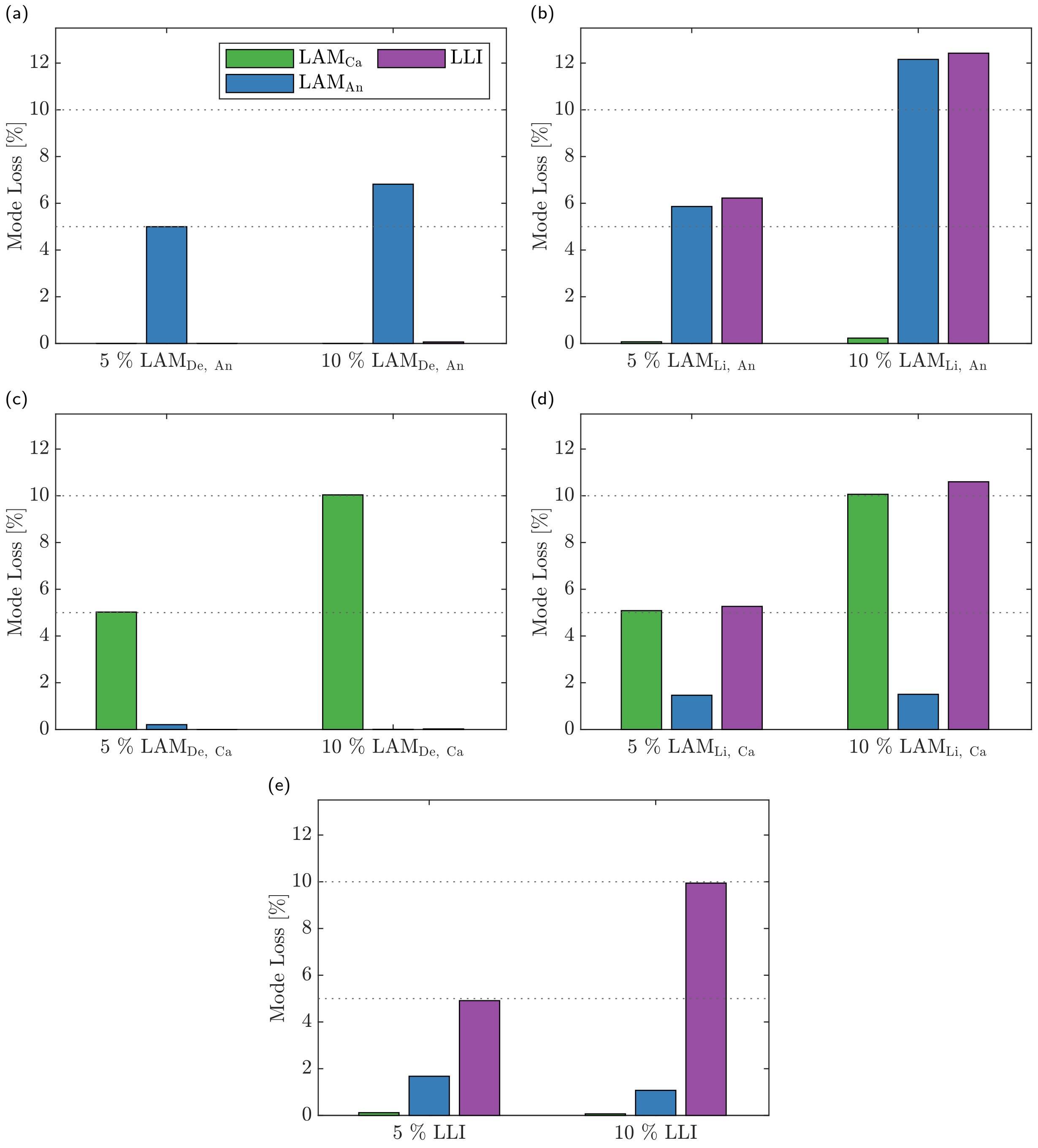

Figure S12: Validation of diagnostic model using synthetic single mode degradation OCV curves; a) Identified degradation modes with 5 and 10 % simulated $\mathrm{LAM_{An,\ Delithiated}}$. b) Identified degradation modes with 5 and 10 % simulated $\mathrm{LAM_{An,\ Lithiated}}$. c) Identified degradation modes with 5 and 10 % simulated $\mathrm{LAM_{Ca,\ Delithiated}}$. d) Identified degradation modes with 5 and 10 % simulated $\mathrm{LAM_{Ca,\ Lithiated}}$. e) Identified degradation modes with 5 and 10 % simulated LLI. In all cases horizontal lines indicate 5 and 10 % mode loss.

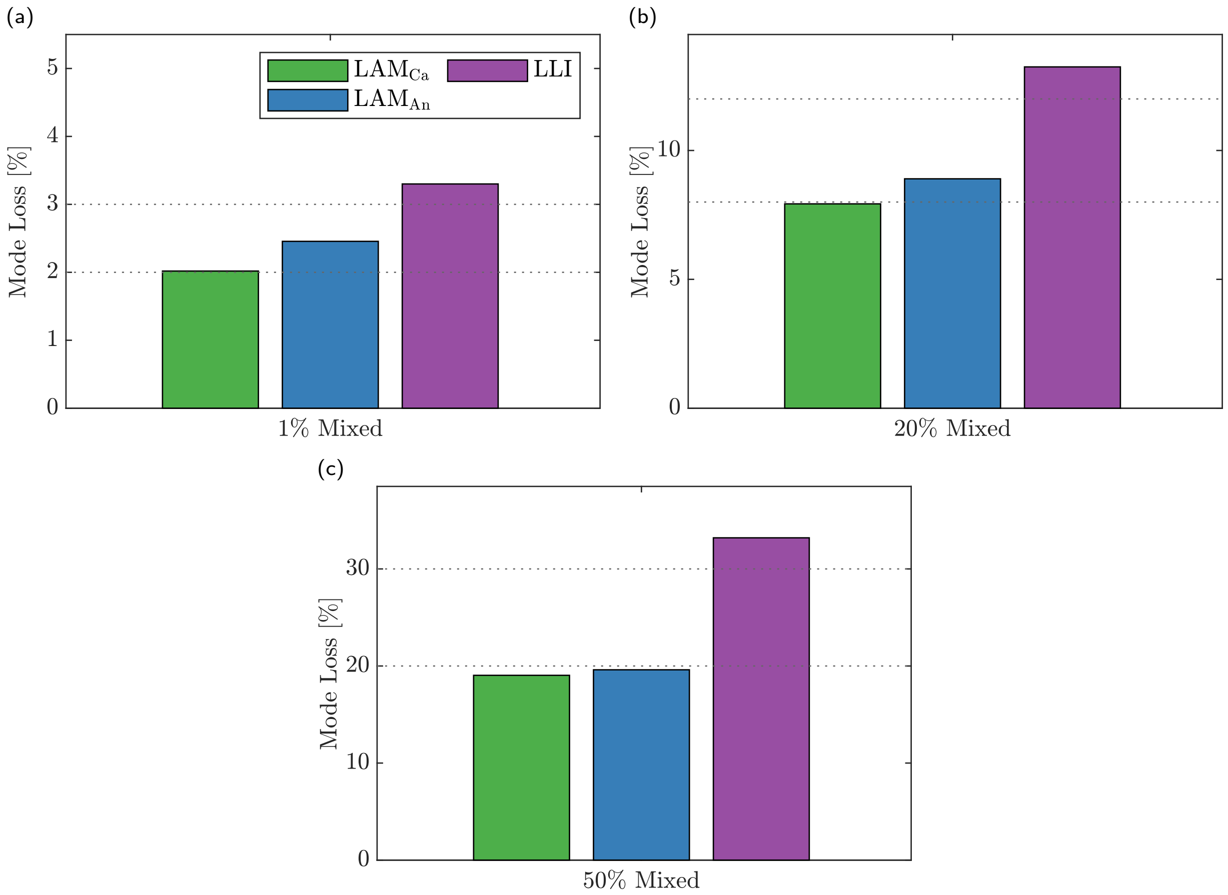

Figure S13: Validation of diagnostic model using synthetic mixed mode degradation OCV curves; a) Identified degradation modes with 1 % degradation in each mode (2 % total LAM_{An} and LAM_{Ca}, 3 % LLI). b) Identified degradation modes with 4 % degradation in each mode (8 % total LAM_{An} and LAM_{Ca}, 12 % LLI). c) Identified degradation modes with 10 % degradation in each mode (20 % total LAM_{An} and LAM_{Ca}, 30 % LLI). Horizontal lines indicate the actual total mode losses in each case.

cell voltage at measurement was 3.78 $\pm$ 0.002 V. The measured spectra are shown in Figure 3 a-f. After acquisition, the impedance spectra were fitted using the fitting model found in Figure S14. The model uses 2 R-CPE pairs to distinguish between anode and cathode charge transfer processes, which are expected to have distinct time constants, with longer time constants contributed by the cathode [12]. Typical uncertainties in fitting were $<\pm$ 2 % for R_0 and R_{CT}, and $<\pm$ 10 % for R_{SEI}. The increased uncertainty in fit for R_{SEI} is expected as there is overlap between the contribution of R_{SEI} and R_{CT}, with R_{SEI} having a significantly smaller magnitude.

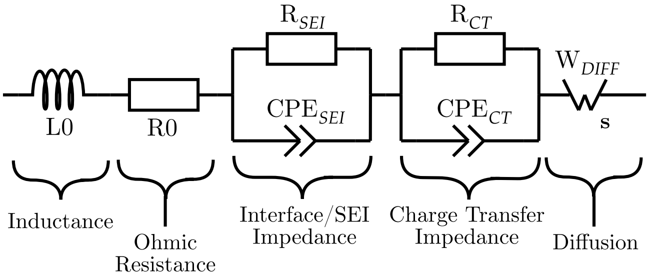

Figure S14: EIS fitting equivalent circuit model.

The changes in each associated resistance for each pack is presented in Figure S15 a-f, showing the normalised increase in R_0, R_{SEI} and R_{CT}. For P20.0/0.0 (Figure S15 a) the magnitude in R_{CT} increased of 4.8 and 3.8 times in B1 and B2 respectively. Meanwhile P45.0/0.0 shows an increases in R_{CT} of 2.6 and 2.3 times, and the divergence between cells reduces at higher temperatures. The trends observed in the homogeneous packs are replicated in the heterogeneous P32.5/+25.0, P32.5/-12.5 and P32.5/+25.0 packs, as shown in Figure S15 b, d and f. In all cases, the change in R_0 and R_{SEI} is negligible compared to the change in the fitted R_{CT} value, confirming that the impedance growth observed is dominated by changes to R_{CT}.

As a final confirmation of the behaviour of each impedance, the normalised resistance growth values for each fitted resistance and all 12 cells studied are presented in Figure S16 a-c for R_0, R_{SEI} and R_{CT}, plotted against the cycling temperature. No significant correlation with temperature is observed in R_0 (Figure S16 a). However, R_{SEI} growth (Figure S16 b) is observed to be clearly a thermally correlated process as expected, with significant increases above 40.0 °C. Meanwhile the rate of growth of R_{CT} (Figure S16 c) is negatively correlated with temperature, the rate of growth halving when cycling temperature increases from 20.0 to 45.0 °C.

2.5. Cell Post-Mortem Analysis

In order to provide independent evidence of the degradation mode identified via OCV fitting, a post-aging teardown of a single cell from each of the homogeneous packs (P20.0/0.0 and P45.0/0.0) was carried out. In both cases, B1 was selected for teardown, and an uncycled cell from the same batch was also disassembled to provide a reference baseline. In each case, the cell was opened in an Argon-filled glovebox, and the electrode stack unwound to reveal the electrodes. Electrodes were harvested from layers 1, 10, 20 and 25 (with 50 total anode/cathode pairs in the pack), and samples cut from the centre of the electrode. No qualitative morphological changes were observed in the electrodes through-thickness, while no significant morphological changes were observed in the harvested anodes compared to the baseline, supporting the previously identified limited anode loss of active material. Washing of the harvested anodes and limitations to the magnification of the instrument used meant it is not possible to assess the degree of SEI layer formation (which would lead to LLI) based on this postmortem.

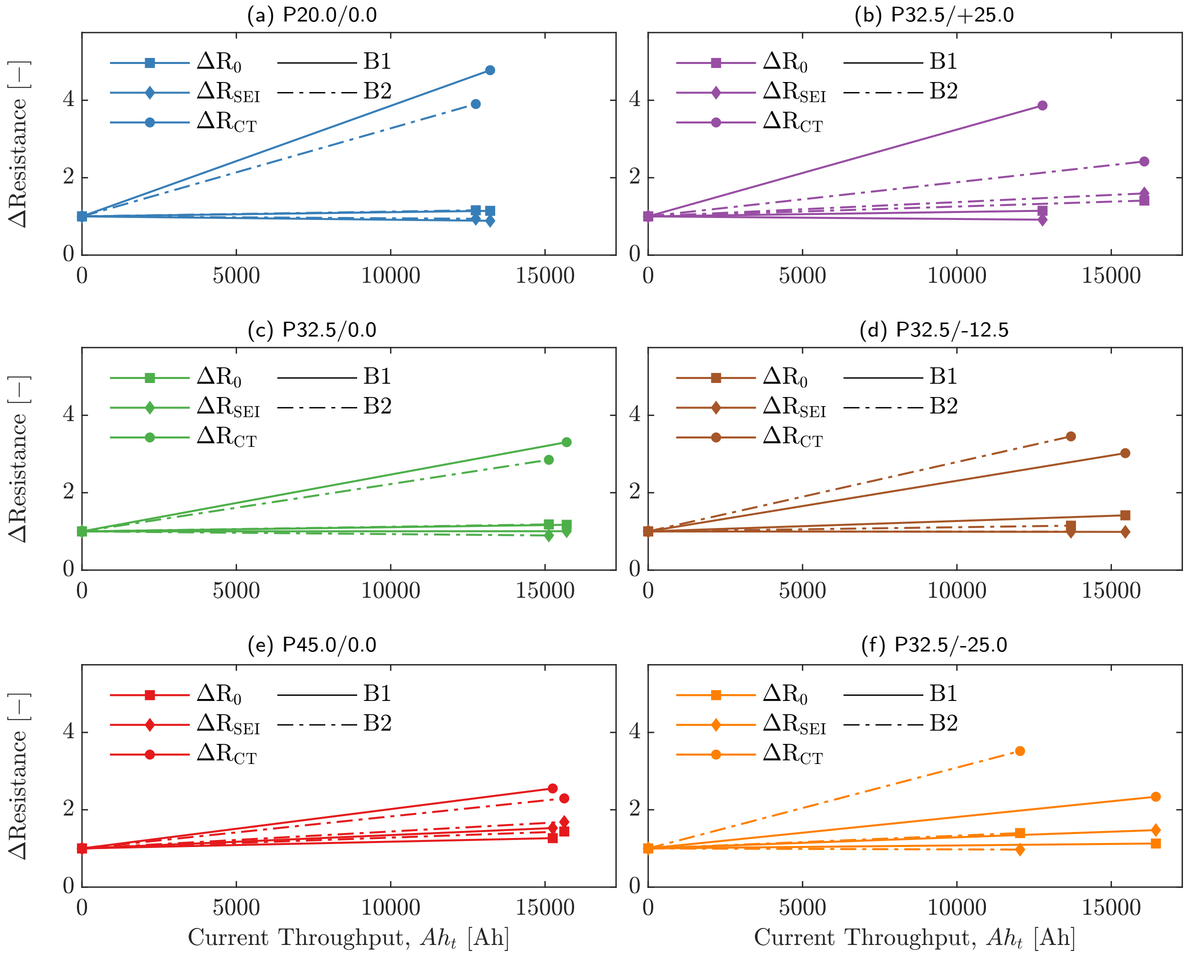

Figure S15: Proportional change in fitted resistances R_0, R_{SEI} and R_{CT}, for all packs. a) P20.0/0.0. b) P32.5/+25.0. c) P32.5/0.0. d) P32.5/-12.5. e) P45.0/0.0. f) P32.5/-25.0.

Figure S17 presents scanning electron microscopy images obtained from samples at the centre of the cathodes. In Figure S17 a and b, for the uncycled cathode, two particle morphologies are observed. The cathode consists of spherical agglomerate particles (NMC) consisting of fine angular particles mixed with layered and faceted grain particles (LCO). Figure S17 c and d show the cathode from P20.0/0.0 B1, and cracks are highlighted in both particle morphologies. Highlighted feature 1 (Figure S17 c) shows intragranular cracks in the faceted particles, extending both through and in the layer planes. Feature 2 also shows evidence of crack formation in the agglomerate particles, while in other locations pit-holes and pulverisation of the agglomerates is observed. In the lower magnification image (Figure S17 d), deposition of debris, likely lost cathode material, is highlighted as feature 3. Considering P45.0/0.0 B1 (Figure S17 e and f), pit-holes and pulverisation of the agglomerate particles is also observed (feature 4), whilst fracture also occurred in the faceted morphology particles (feature 5). Deposition of debris in the binder is also observed in the low-magnification images (feature 6). This debris is likely lost active material from the cathode electrode particles, released during particle fracture and pulverisation. As this postmortem analysis is only able to qualitatively identify electrode morphological damage, it is not possible to qualitatively assess the relative amount of loss of cathode active material in each case. However, qualitatively, no significant differences were observed between harvested cathodes from cells cycled at low and high temperatures, supporting the results from OCV fitting that the

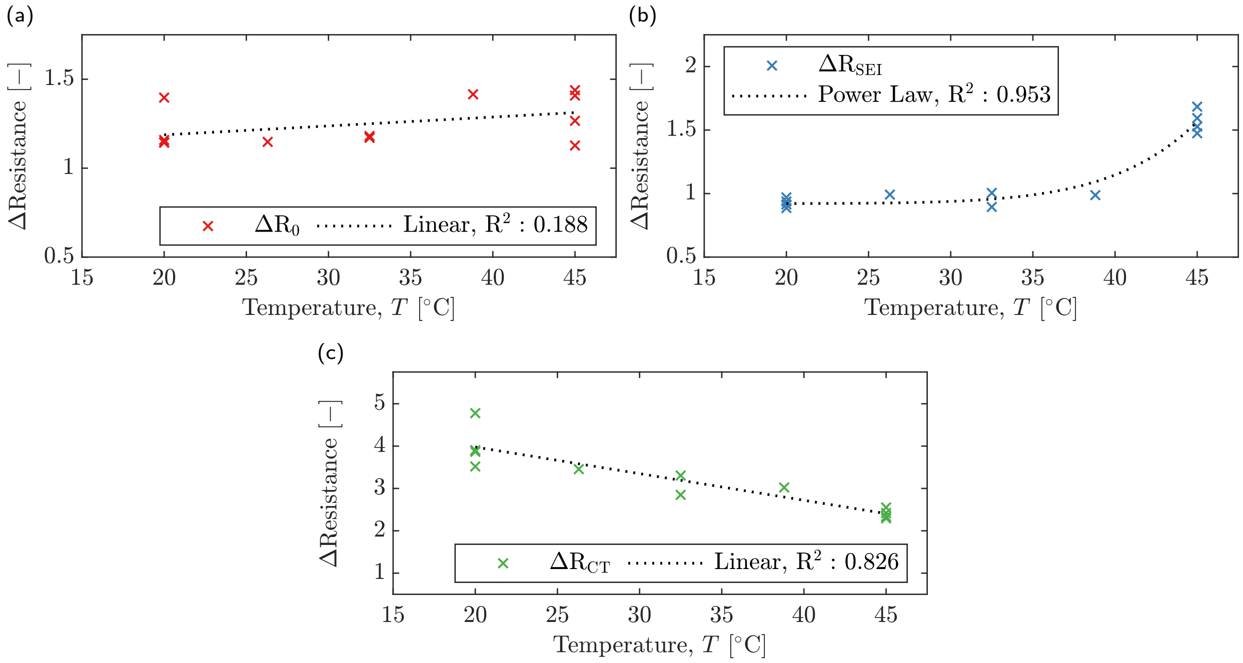

Figure S16: Proportional change in fitted resistances R_0, R_{SEI} and R_{CT} against temperature for all cells. b) R_0. a) R_{SEI}. c) R_{CT}.

amount of cathode active material loss is relatively independent of the cycling temperature.

3. Supplementary Notes

3.1. Missing Data

As previously mentioned, power failures led to loss of a small portion of the tracked current distribution data. This missing data is tabulated in Table S2.

Table S2: Missing cycling cell current data per pack

Pack	Missing Cycle Numbers	Number Missing	Missing Fraction [%]
P20.0/0.0	341-343	2	0.1
P45.0/0.0	341-343	2	0.1
P32.5/-+25.0	206-219, 296-306	23	1.2
P32.5/0.0	204-216, 294-304, 551-565	38	1.9
P32.5/-25.0	148-160, 241-244	15	0.8
P32.5/-12.5	148-160, 237-240, 558-560	17	0.9

References

[1] K. Rumpf, M. Naumann, A. Jossen, Experimental investigation of parametric cell-to-cell variation and correlation based on 1100 commercial lithium-ion cells, Journal of Energy Storage 14 (2017) 224–243. URL: `https://doi.org/10.1016/j.est.2017.09.010`. doi:10.1016/j.est.2017.09.010.

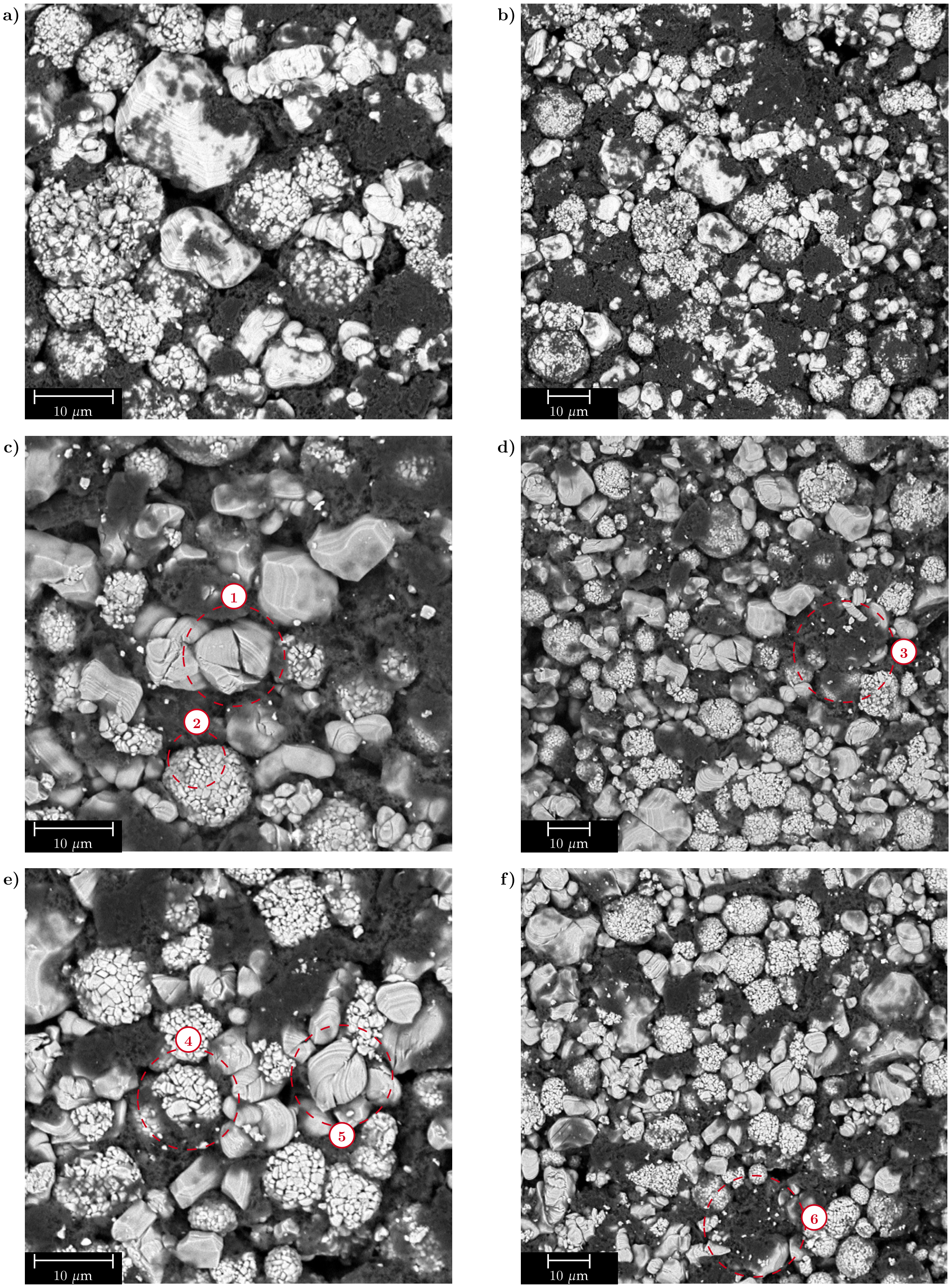

Figure S17: SEM images of cathodes from fresh and cycled cells, with features highlighted. All samples taken from the centre of cathode No. 25 (centre of electrode stack). 20 °C cycled cell selected is P20.0/0.0 B1, 45 °C cycled cell selected was P45.0/0.0 B1 a) Fresh cathode. b) Fresh cathode (low magnification). c) Cathode cycled at 20 °C. d) Cathode cycled at 20 °C. (low magnification). e) Cathode cycled at 45 °C. f) Cathode cycled at 45 °C. (low magnification). Highlighted features: 1) Fracture in LCO particle. 2) Fracture in NMC particle. 3) Debris deposited in binder. 4) Damage to NMC particle. 5) Fracture of LCO particle. 6) Debris deposited in binder.

[2] Y. Troxler, B. Wu, M. Marinescu, V. Yufit, Y. Patel, A. J. Marquis, N. P. Brandon, G. J. Offer, The effect of thermal gradients on the performance of lithium-ion batteries, Journal of Power Sources 247 (2014) 1018–1025. doi:10.1016/j.jpowsour.2013.06.084.

[3] I. A. Hunt, Y. Zhao, Y. Patel, J. Offer, Surface Cooling Causes Accelerated Degradation Compared to Tab Cooling for Lithium-Ion Pouch Cells, Journal of The Electrochemical Society 163 (2016) A1846–A1852. doi:10.1149/2.0361609jes.

[4] A. Hales, L. B. Diaz, M. W. Marzook, Y. Zhao, Y. Patel, G. Offer, The Cell Cooling Coefficient: A Standard to Define Heat Rejection from Lithium-Ion Batteries, Journal of The Electrochemical Society 166 (2019) A2383–A2395. doi:10.1149/2.0191912jes.

[5] M. Dubarry, C. Truchot, B. Y. Liaw, Synthesize battery degradation modes via a diagnostic and prognostic model, Journal of Power Sources 219 (2012) 204–216. URL: `http://dx.doi.org/10.1016/j.jpowsour.2012.07.016`. doi:10.1016/j.jpowsour.2012.07.016.

[6] C. R. Birkl, E. McTurk, M. R. Roberts, P. G. Bruce, D. A. Howey, A Parametric Open Circuit Voltage Model for Lithium Ion Batteries, Journal of The Electrochemical Society 162 (2015) A2271–A2280. doi:10.1149/2.0331512jes.

[7] C. R. Birkl, M. R. Roberts, E. Mcturk, P. G. Bruce, D. A. Howey, Degradation diagnostics for lithium ion cells, Journal of Power Sources 341 (2017) 373–386. URL: `http://dx.doi.org/10.1016/j.jpowsour.2016.12.011`. doi:10.1016/j.jpowsour.2016.12.011.

[8] R. Jung, M. Metzger, F. Maglia, C. Stinner, H. A. Gasteiger, Oxygen Release and Its Effect on the Cycling Stability of LiNi x Mn y Co z O 2 (NMC) Cathode Materials for Li-Ion Batteries, Journal of The Electrochemical Society 164 (2017) A1361–A1377. doi:10.1149/2.0021707jes.

[9] C. Sole, N. E. Drewett, L. J. Hardwick, Insitu Raman study of lithium-ion intercalation into microcrystalline graphite, Faraday Discussions 172 (2014) 223–237. doi:10.1039/c4fd00079j.

[10] C. S. Kim, K. M. Jeong, K. Kim, C. W. Yi, Effects of capacity ratios between anode and cathode on electrochemical properties for lithium polymer batteries, Electrochimica Acta 155 (2015) 431–436. doi:10.1016/j.electacta.2014.12.005.

[11] C. Mao, R. E. Ruther, J. Li, Z. Du, I. Belharouak, Identifying the limiting electrode in lithium ion batteries for extreme fast charging, Electrochemistry Communications 97 (2018) 37–41. doi:10.1016/j.elecom.2018.10.007.

[12] J. Zhu, M. Knapp, X. Liu, P. Yan, H. Dai, X. Wei, H. Ehrenberg, Low temperature separating lithium-ion battery interfacial polarization based on distribution of relaxation times (DRT) of impedance, IEEE Transactions on Transportation Electrification (2020) 1–1. doi:10.1109/tte.2020.3028475.
